# News Items, Medical Facts, &c.

**Published:** 1859-01

**Authors:** 


From the London Lancet.

### NEWS ITEMS, MEDICAL FACTS, &C.

*Death of an English Surgeon.*—The "Overland Friend of China," in announcing the death of Dr. Aurelius Harland, which took place at Hongkong on the 12th of September, of congestion of the brain, pays an animated tribute to his memory. That paper says:—"No event in Hongkong since the death of John Robert Morrison in 1843, described by Henry Pottinger as a public calamity, has created such a deep and general sensation as the death of our esteemed friend Dr. Harland. Dr. Harland was a resident here of fourteen years' standing. For a considerable period after his arrival, he devoted himself to the study of the Chinese language, with a view at first to travel in the interior. But little heed was given to the acquisition of wealth by medical practice. Not long after his arrival he accepted the post of resident surgeon at the Seamen's Hospital, and there he spent many happy years, having leisure sufficient to pursue, to some extent, the sciences to which he was so much attached, hardly any branch escaping his unwearied ken. But he never allowed these to interfere with his medical duties, and he acted not only as the physician, but as the friend, wherever it was needed. Dr. Harland may be looked upon as a martyr to his professional zeal. Many years ago he attended a number of Chinamen in the employ of a shipwright here, who lived in a very unhealthy locality, and were dying in numbers of that fearful fever which in those times was so prevalent on this island. With these unfortunate men he sat up night after night, and there himself was attacked with a severe form of remittent fever, ever since which he has been subject to the return of the disease at longer or shorter intervals. One of these attacks has deprived the colony of the most universally respected and talented practitioner it has ever possessed." H. F. H. Hance, Ph. D., of Hongkong, has also published a biographical sketch of the deceased, from which the following are extracts: "Dr. Harland was the son of a physician at Scarborough, Yorkshire, who still lives to lament the loss. He graduated in 1844 at Edinburgh University, where he bore away many honors, was looked on as the foremost medical student of the day, and was elected member of various learned societies. Soon after passing the London Royal College of Surgeons he came out to Hongkong, in which he spent eleven years, previous to his recent visit to England. While here he acquired a reading knowledge of Mandarin, and devoted much time to the study of Chinese medicine and physiology, communicating some of the results of his labors to the public through the Journal of the China branch of the Royal Asiatic Society. The prospective opening of China by the treaty of Tien-tsin caused him the liveliest satisfaction, as likely to facilitate the carrying out of a long cherished project—the scientific exploration of some of the less known portions of the empire. There are few who were better fitted to execute such a task. For some time past he had been carefully and steadily collecting materials for a comparative study of the natural productions of Hongkong, which he regarded as one of the most important of his self-imposed tasks." "After Dr. Harland's death," Dr. Hance adds in a private letter, "the Government here sent round a circular notifying his decease, and expressing the deepest regret at the loss of so valuable a servant, and a hope that all officers would attend his remains to the grave. This was a most unusual official document, and may be considered as a proof of the esteem in which he was held."

